# Differences in the DNA methylome of T cells in adults with asthma of varying severity

**DOI:** 10.1186/s13148-024-01750-7

**Published:** 2024-10-08

**Authors:** Yixuan Liao, Raymond G. Cavalcante, Jonathan B. Waller, Furong Deng, Anne M. Scruggs, Yvonne J. Huang, Ulus Atasoy, Yahong Chen, Steven K. Huang

**Affiliations:** 1 Department of Pulmonary and Critical Care Medicine, Peking University Third Hospital, No.49, Huayuan North Road, Haidian District, Beijing, 100191 China; 2 Department of Pulmonary and Critical Care Medicine, Beijing Hospital, National Center of Gerontology, Institute of Geriatric Medicine, Chinese Academy of Medical Sciences, Beijing, China; 3 Department of Computational Medicine and Bioinformatics, University of Michigan, Ann Arbor, MI USA; 4 Division of Pulmonary and Critical Care Medicine, Department of Internal Medicine, University of Michigan, 6301 MSRB III, 1150 W Medical Center Dr., Ann Arbor, MI 48109 USA; 5 Department of Occupational and Environmental Health Sciences, School of Public Health, Peking University, Beijing, China; 6 Division of Allergy and Clinical Immunology, Department of Internal Medicine, University of Michigan, Ann Arbor, MI USA

**Keywords:** DNA methylation, Epigenetics, Fraction of exhaled nitric oxide

## Abstract

**Background:**

DNA methylation plays a critical role in asthma development, but differences in DNA methylation among adults with varying asthma severity are less well-defined.

**Objective:**

To examine how DNA methylomic patterns differ among adults with asthma based on asthma severity and airway inflammation.

**Methods:**

Peripheral blood T cells from 35 adults with asthma in Beijing, China, were serially collected over time (130 samples total) and analyzed for global DNA methylation using the Illumina MethylationEPIC Array. Differential methylation was compared among subjects with varying airway inflammation and severity, as measured by fraction of exhaled nitric oxide, forced expiratory volume in one second (FEV1), and Asthma Control Test (ACT) scores.

**Results:**

Significant differences in DNA methylation were noted among subjects with different degrees of airway inflammation and asthma severity. These differences in DNA methylation were annotated to genes that were enriched in pathways related to asthma or T cell function and included gene ontology categories related to MHC class II assembly, T cell activation, interleukin (IL)-1, and IL-12. Genes related to P450 drug metabolism, glutathione metabolism, and developmental pathways were also differentially methylated in comparisons between subjects with high vs low FEV1 and ACT. Notable genes that were differentially methylated based on asthma severity included *RUNX3*, several members of the *HLA* family, *AGT*, *PTPRC*, *PTPRJ*, and several genes downstream of the *JAK2* and *TNF* signaling pathway.

**Conclusion:**

These findings demonstrate how adults with asthma of varying severity possess differences in peripheral blood T cell DNA methylation that contribute to differences in clinical indices of asthma.

**Supplementary Information:**

The online version contains supplementary material available at 10.1186/s13148-024-01750-7.

## Background

DNA methylation is a fundamental epigenetic mechanism that plays a critical role in regulating gene expression and the development of many diseases, including asthma. Multiple studies have demonstrated that changes in DNA methylation are associated with asthma development [1–7] and include genes such as *RUNX3* [8], *IL13* [8], *NOS2* [9], *ARG1* [10], and *ALOX12* [11] that highlight the importance of these genes and the role of DNA methylation changes in asthma pathogenesis. Global differences in DNA methylation patterns have also been demonstrated in cord blood [1, 2], peripheral blood mononuclear cells [8], and nasal epithelium [12] of children with asthma. Many of these changes occur early in development and support the fetal-origin hypothesis of the disease.

Although differences in DNA methylation are well-described in children with asthma, the role of DNA methylation changes in adults is less appreciated. Most of the studies that link DNA methylation to asthma focus on how DNA methylation changes contribute to asthma development, but less is known regarding how methylation changes may affect an individual’s severity, including the degree of lung function impairment, airway obstruction, or asthma control. As asthma is heterogeneous, the role of DNA methylation in affecting different clinical indices of asthma is less clear. The implications of this, however, are important as methylation changes have been associated with drug response and can inform future strategies in precision medicine [13]. Finally, as different cell types exhibit unique patterns of methylation, identifying methylation changes within a specific cell population, as compared to other studies that utilize whole blood, may provide better insight into the biological significance of these methylation changes [7, 14].

In this study, we sought to examine how the patterns of DNA methylation within the peripheral blood T cells of adult asthmatics differ among patients with different severities, as measured by lung function (baseline forced expiratory volume in 1 s [FEV1]), symptom control (Asthma Control Test [ACT]), and airway inflammation, as measured by fraction of exhaled nitric oxide (FeNO). In addition, given that most studies of asthma and DNA methylation were conducted in European and North American countries that may have higher rates of certain genomic variants [15], we sought to utilize a cohort from Beijing, China, to assess the relationship between DNA methylation and asthma among other populations in other countries. Ultimately, the differences in DNA methylation that we observed support epigenetic changes as a potential mechanism for affecting the airway inflammation, symptoms, and overall severity of adults with asthma.

## Methods

### Patient subjects

Patients > 18 years of age who lived in Beijing, China, with a clinical diagnosis of asthma (as defined by clinician assessment of individuals with a history of episodic symptoms of dyspnea, cough, or wheezing, a positive methacholine challenge test, and exclusion of other respiratory conditions that may mimic asthma) were recruited to participate in the study. Inclusion criteria included either a positive bronchodilation test with an increase in FEV1 of 12% and 200 ml from baseline after 400 µg albuterol inhalation or a positive methacholine challenge test with a decrease in FEV1 of 20% from baseline after methacholine inhalation with concentration < 4 mg/ml. Patients were required to be free of asthma exacerbation for at least one month prior to enrollment. Patients who were current smokers or had a greater than 5-pack-year smoking history or have a history of chronic lung disease other than asthma (including COPD, lung cancer, active tuberculosis, or interstitial lung disease) were excluded. All patients had demographic information, asthma history, spirometry, measures of FeNO, and assessment of asthma symptoms, as measured by ACT, taken at baseline. Patients were followed longitudinally up to every month for 12 months. At follow-up visits, spirometry including FEV1, FeNO, and ACT was measured monthly and blood samples were taken at 0, 3, 6, 9, and 12 months of follow-up. None of the patients had a recent asthma exacerbation or severe illness at the time the samples were taken. All patients provided written informed consent, and the study was approved by both the Peking University and University of Michigan Institutional Review Board.

### Isolation of peripheral blood CD3 + T cells

Peripheral blood CD3 + T cells were isolated using anti-human CD3 magnetic nanoparticles and a cell separation magnet. Briefly, blood was first centrifuged and peripheral blood mononuclear cells were isolated using Ficoll-Paque PLUS (Cytiva, Cat No. 17144002, Marlborough, MA) and resuspended in PBS. Cells were then labeled with anti-human CD3 Particles DM (Cat. No. 552593, BD Biosciences, Franklin Lakes, NJ) and separated using the Cell Separation Magnet (Cat. No. 552311, BD Biosciences).

### DNA isolation and methylation analysis

Global DNA methylation patterns were analyzed using the Infinium MethylationEPIC array (Illumina, Inc, San Diego, CA), which interrogates over 850,000 methylation sites at single-nucleotide resolution. DNA was isolated using the QlAmp DNA Micro Kit (Cat No. 56304, QIAGEN, Germany) and stored in − 80 °C before being shipped to the United States for bulk processing. DNA was quantitated using the Qubit high sensitivity DNA assay (ThermoFisherScientific, Waltham, MA) and assessed for quality using the TapeStation genomic DNA kit (Agilent, Santa Clara, CA). For each sample, 250 ng of genomic DNA was bisulfite converted using Zymo EZ DNA Methylation kit (Zymo Research, Irvine, CA) before being hybridized to the Infinium MethylationEPIC BeadChip array for analysis. All raw data from the EPIC array were deposited in the National Center for Biotechnology Information Gene Expression Omnibus database under accession number GSE226257.

### Bioinformatics and statistical analysis

All codes used to format, clean and plot the data are publicly available in GitHub and are available upon request. Raw red/green IDAT files were read into R using the minfi Bioconductor package (v1.32.0) [16]. Initial quality control based on detection *p* values and signal intensity were performed using the ENmix Bioconductor package (v1.22.4) [17]. Probe intensities were background- and dye-corrected using the NOOB background correction [18] followed by stratified quantile normalization. Probes with a detection *p* value > 0.05 in more than 5% of the samples were removed from analysis. By this criterion, 11,936 CpGs were removed from analysis. If more than 5% of the probes in a sample had a detection *p* value > 0.05, the sample would be removed from analysis. None of the samples met this criterion. Any known cross-hybridizing probes [19, 20], any probe within 2 base pairs of a single-nucleotide polymorphism, and probes from chromosome X and Y were also excluded.

Probes were tested for differential methylation using the limma R Bioconductor package (v3.38.3) using linear models whose standard errors were moderated using an empirical Bayes model [21]. To assess the relationship between airway inflammation, asthma severity, and DNA methylation, methylation levels were analyzed against the subjects’ FeNO, FEV1, and ACT after adjustment for covariates. Since most subjects provided multiple samples over time, adjustments were made for repeat measures taken longitudinally from the same subject. Although samples were isolated for CD3 + T cells, we additionally used the FlowSorted.Blood.EPIC package (v1.4.1) to deconvolute samples into constituent cell types (CD8 + T cells, CD4 + T cells, NK cells, B cells, monocytes, and neutrophils) using a modified version of the Houseman method [22] and these estimates were also used as covariates in the model. The following models were fit for each measure of asthma (FeNO, FEV1, and ACT), where repeated sampling was accounted for with the subject_id, and the blood cell-type deconvolution proportions are continuous covariates:

$$\begin{gathered} \sim 0 + \left\{ {{\text{asthma}}\;{\text{measure}}} \right\} + {\text{subject}}\_{\text{id}} + {\text{sex}} + {\text{CD8T}} \hfill \\ \quad\;\;\; + {\text{CD4T}} + {\text{NK}} + {\text{Bcell}} + {\text{Mono}} + {\text{Neu}} \hfill \\ \end{gathered}$$

Measures of FeNO, FEV1, and ACT were initially assessed as continuous variables, but because patients’ inflammation and asthma severity did not significantly change over time (Additional file 1: Figure S2), we binned subjects into top 40% and bottom 40% of each measure based on their mean measures over time.

A difference in methylation (beta value) of  ≥ 5% and adjusted *p* value of < 0.05 (adjusted to ensure a false discovery rate < 5%) was deemed statistically significant. Differentially methylated probes were annotated to genic regions and CpG islands, shores, or shelves using the annotatr R Bioconductor package (v1.7.3) [23]. Methylation significance and degree of differential methylation was then summarized at the gene level. A gene was considered differentially methylated and statistically significant if the adjusted *p* value for any of the CpG sites within the gene was < 0.05. The methylation difference for a gene was then calculated as the weighted average of the methylation difference across all probes in a gene, with methylation values for each probe weighted by the -log10(*p* value) for the given probe. This allows those probes with lower *p* values to account for a greater degree of differential methylation and allowed us to identify differentially methylated genes while adjusting, at least partly, to the uneven distribution of CpGs on different genes, the variable number of CpG sites for different genes, and potential biases within the Epic array itself. The functional significance of differentially methylated genes was analyzed using iPathway Guide (Advaita Bioinformatics, Ann Arbor, MI) to identify enrichment of gene ontology (GO) categories and Kyoto Encyclopedia of Genes and Genomes (KEGG) pathways and was analyzed over a denominator of all genes assayed by the array. Networks were constructed from significant genes within pathways using iPathway Guide.

## Results

Thirty-seven adult asthmatics from Beijing, China, were initially screened. As two subjects did not provide blood samples, 35 subjects were included in the analysis and their baseline characteristics are shown in Table 1. The mean age of the subjects was 42 years, with the mean reported age of asthma onset of 36.9 years. Patients were followed longitudinally for up to a year, and blood samples from subjects were taken every three months when available. From 35 participants, 130 blood specimens were collected and used for DNA methylomic analysis (Additional file 1: Figure S1A). Although we specifically isolated T cells using anti-CD3 magnetic beads for DNA methylomic analysis, cell-type deconvolution based on the DNA methylation patterns was also performed, which confirmed that the DNA was mostly derived from CD4 + and CD8 + (CD4 > CD8) T cells as compared to NK, B cells, monocytes, and neutrophils (Additional file 1: Figure S1B).

**Table 1:** Demographic and clinical characteristics of participants at baseline

Age, yr	
Mean ± SD	42 ± 11.6
Reported age of asthma onset, y	
Mean ± SD	36.9 ± 11.3
Reported gender	
Male, n (%)	20 (57.1)
Female, n (%)	14 (40.0)
Not reported, n (%)	1 (2.9)
Inferred biologic sex based on methylation	
Male, n (%)	21 (60.0)
Female, n (%)	14 (40.0)
BMI, kg/m^2^	
Mean ± SD	25.1 ± 4.0
Pulmonary function	
FEV1 (% predicted)	
Mean ± SD	75.6 ± 22.3
FVC (% predicted)	
Mean ± SD	89.3 ± 18.1
FEV1/FVC (%)	
Mean ± SD	69.2 ± 13.3
Asthma control test score	
Mean ± SD	18.8 ± 4.3
Fraction of exhaled nitric oxide (ppb)	
Mean	48.6 ± 35.3
Medications	
ICS only, n (%)	4 (11.4)
ICS + LABA, n (%)	30 (85.7)
Montelukast, n (%)	25 (71.4)
Oral corticosteroids, n (%)	0 (0)
Co-morbidities	
Allergic rhinitis, n (%)	24 (68.6)
Hypertension, n (%)	4 (11.4)

*BMI* body mass index, *FEV1* forced expiratory volume in one second, *FVC* forced vital capacity, *ICS* inhaled corticosteroid, *LABA* long acting beta agonist

### Stability of DNA methylation and clinical indices of FEV1, FeNO, and ACT over time

One of our initial goals was to determine whether DNA methylation might change as asthma symptoms change over time for each subject. We also sought to determine whether there were linear correlations between clinical indices of asthma and overall patterns of DNA methylation. However, longitudinal follow-up of the cohort revealed that levels of FEV1, FeNO, and ACT scores remained, for the most part, relatively stable for most subjects throughout the study (Additional file 1: Figure S2). In fact, none of the subjects developed acute exacerbations of their asthma (defined by hospitalizations or need for systemic corticosteroids) that would cause large shifts in FEV1, ACT, or FeNO. Thus, when we initially attempted to associate DNA methylation patterns with absolute values of FEV1, FeNO, and ACT, statistical modeling demonstrated a poor correlation between DNA methylation and changes in subjects’ FEV1, FeNO, or ACT over time, with no differences in methylation that met the false discovery threshold of less than 0.05. In fact, when we examined the methylation patterns of all the samples from all patients at all time points, principal components analysis shows that there are greater differences in methylation between patients than in the multiple blood samples taken from each individual patient over time (Additional file 1: Figure S3). These data show that the variation in DNA methylation within subjects was less compared to the variation that we observed across subjects.

### Differential methylation associated with differences in FeNO, baseline FEV1, and ACT

**Fig. 1:**
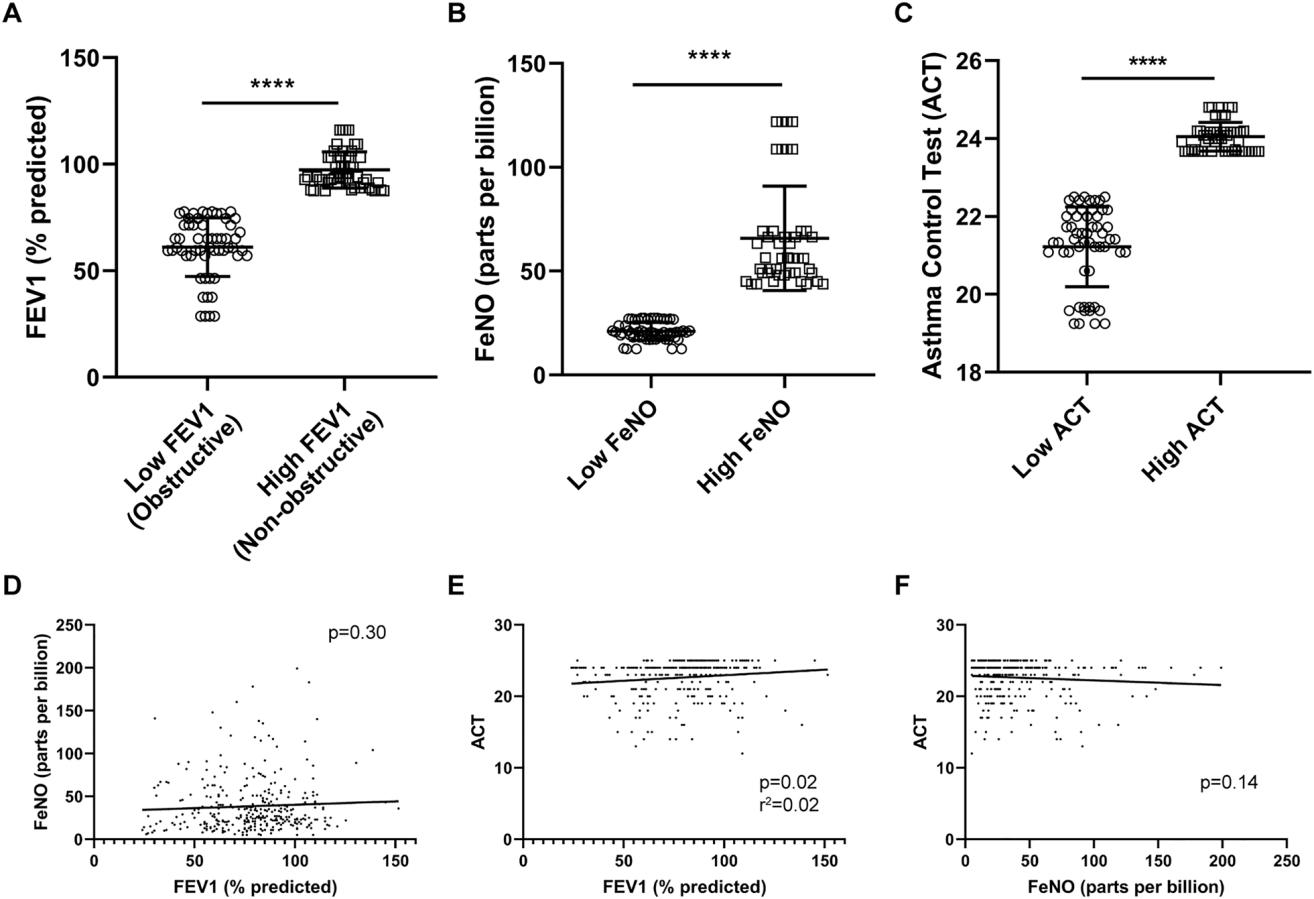
Comparison of forced expiratory volume in 1 s (FEV1), fraction of exhaled nitric oxide (FeNO), and asthma control test (ACT) score among study subjects. (**A**–**C**) Levels of subjects’ pre-bronchodilator FEV1, FeNO, and ACT from the bottom 40% of the cohort (low) were compared to those in the top 40% of the cohort (high). **** *p* < 0.0001. (**D**–**F**) Comparisons were made between subjects’ FEV1 and FeNO (**D**), FEV1 and ACT (**E**), and FeNO and ACT (**F**). Linear regression analysis was performed with p values shown

Although initial attempts to linearly correlate DNA methylation with absolute levels of FeNO, FEV1 and ACT scores were unrevealing, we did note that the cohort consisted of asthmatics with a broad range of underlying severity and control. When each subject’s FEV1, FeNO, or ACT was examined over the entire period of study, a clear segregation was noted among those at the top 40% and those at the bottom 40% of the cohort. Those in the bottom 40% of pre-bronchodilator FEV1 demonstrated an FEV1 consistently less than 80% of predicted (defined clinically as obstructive physiology) as compared to those whose FEV1 was greater than 80% of predicted (non-obstructive) (Fig. 1A). Similarly, those in the top 40% of FeNO had an FeNO greater than 25 parts per billion (ppb), which is often associated with a type 2 high asthma, versus those with low FeNO (< 25 ppb) (Fig. 1B). The same could be used to define those with high ACT scores (considered well-controlled) versus those with lower ACT (Fig. 1C). Those subjects with high FeNO (or high FEV1 or high ACT) demonstrated levels of FeNO (or FEV1 or ACT) that remained high throughout the study and vice versa. Our cohort could thus be segregated into populations of either high vs low FeNO, high vs low FEV1 (non-obstructive vs obstructive lung function), or high vs low ACT. Finally, measures of FeNO, FEV1, and ACT correlated poorly with one another (Fig. 1D–F), indicating that each of these measurements represent distinct, non-overlapping measures of airway inflammation, asthma severity, and control, respectively. Subjects with high FeNO, for example, did not necessarily demonstrate diminished lung function or poor asthma control.

**Fig. 2:**
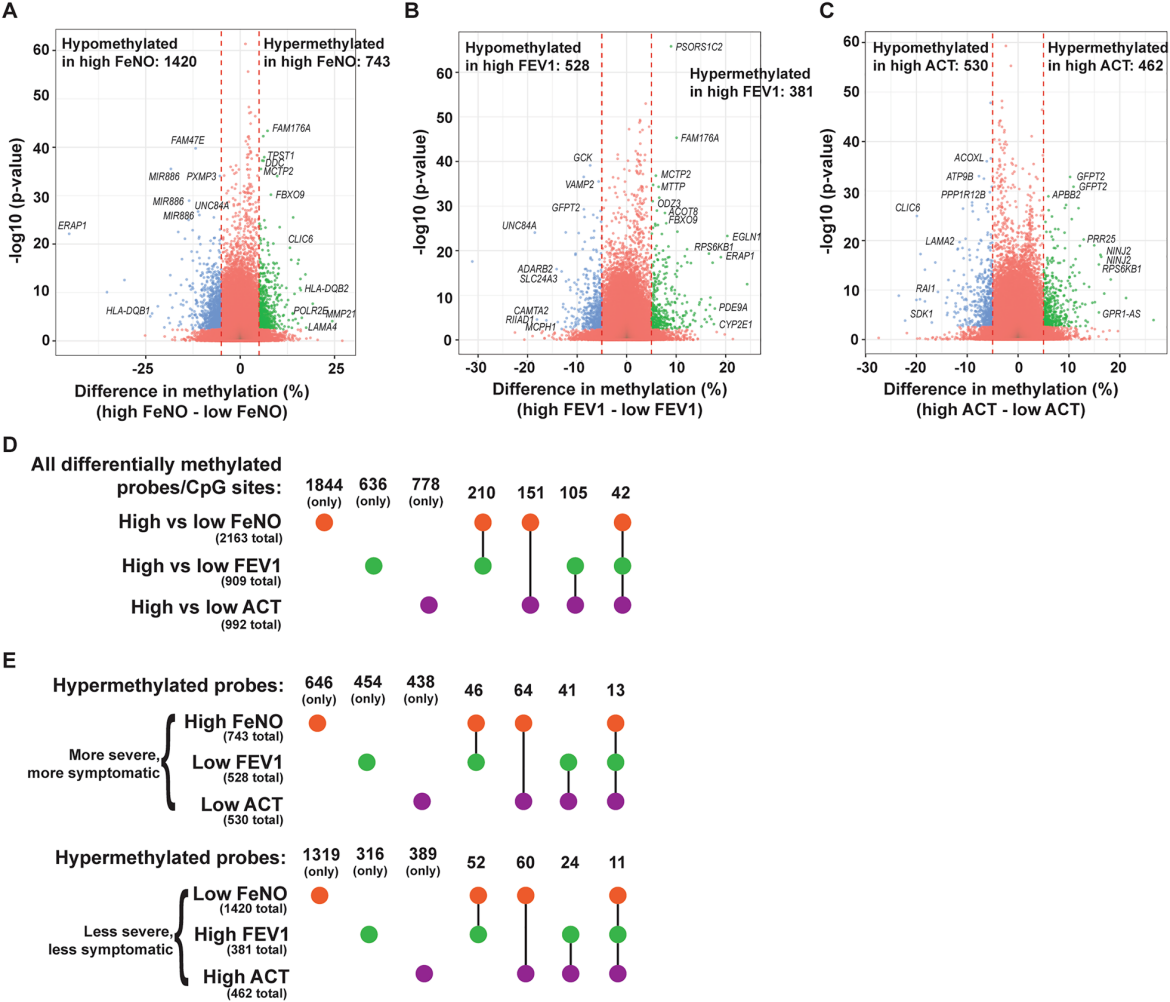
Number of differentially methylated probes/CpG sites between comparisons of high vs low FeNO, high vs low FEV1, and high vs low ACT. (**A**–**C**) Differences in DNA methylation between the high and low FeNO group (**A**), high and low FEV1 group (**B**), and high and low ACT group (**C**) were plotted as a volcano plot against − log10 of the *p* value. Some of the top differentially methylated probes by either p value or difference in methylation were annotated to genes and the gene symbols are indicated in italics. (**D**) UpSet plots were generated from the number of differentially methylated probes or CpG sites among all comparisons and the number of probes that were found in overlap between the three comparisons. (**E**) Upset plots were generated listing the number of overlapping and non-overlapping probes that were hypermethylated among the high (vs low) FeNO group, low (vs high) FEV1 group, and low (vs high) ACT, which we collectively defined as those having a more severe clinical index of asthma. Similar UpSet plots were generated from the number of overlapping and non-overlapping hypermethylated probes from the low (vs high) FeNO, high (vs low) FEV1, and high (vs low) ACT groups, which are defined as individuals with a less severe, less symptomatic disease

Using these dichotomous groupings, we compared the DNA methylation profiles among samples from patients with high vs low FeNO, FEV1, and ACT. Adjusted for repeat sampling, this analysis identified many differentially methylated CpG sites, this time that were statistically significant by adjusted *p* value, between high vs low FeNO, high vs low FEV1, or high vs low ACT (Fig. 2A–C). Many of the top differentially methylated sites were annotated to genes, as shown. Only a small percentage of the differentially methylated CpG sites for a given comparison was found in common to be differentially methylated in another comparison (Fig. 2D and E), indicating that each of these distinct clinical parameters (FeNO, FEV1, or ACT score) was associated with a different set of differentially methylated CpG sites.

### Annotation of differential methylation sites to genic regions

**Fig. 3:**
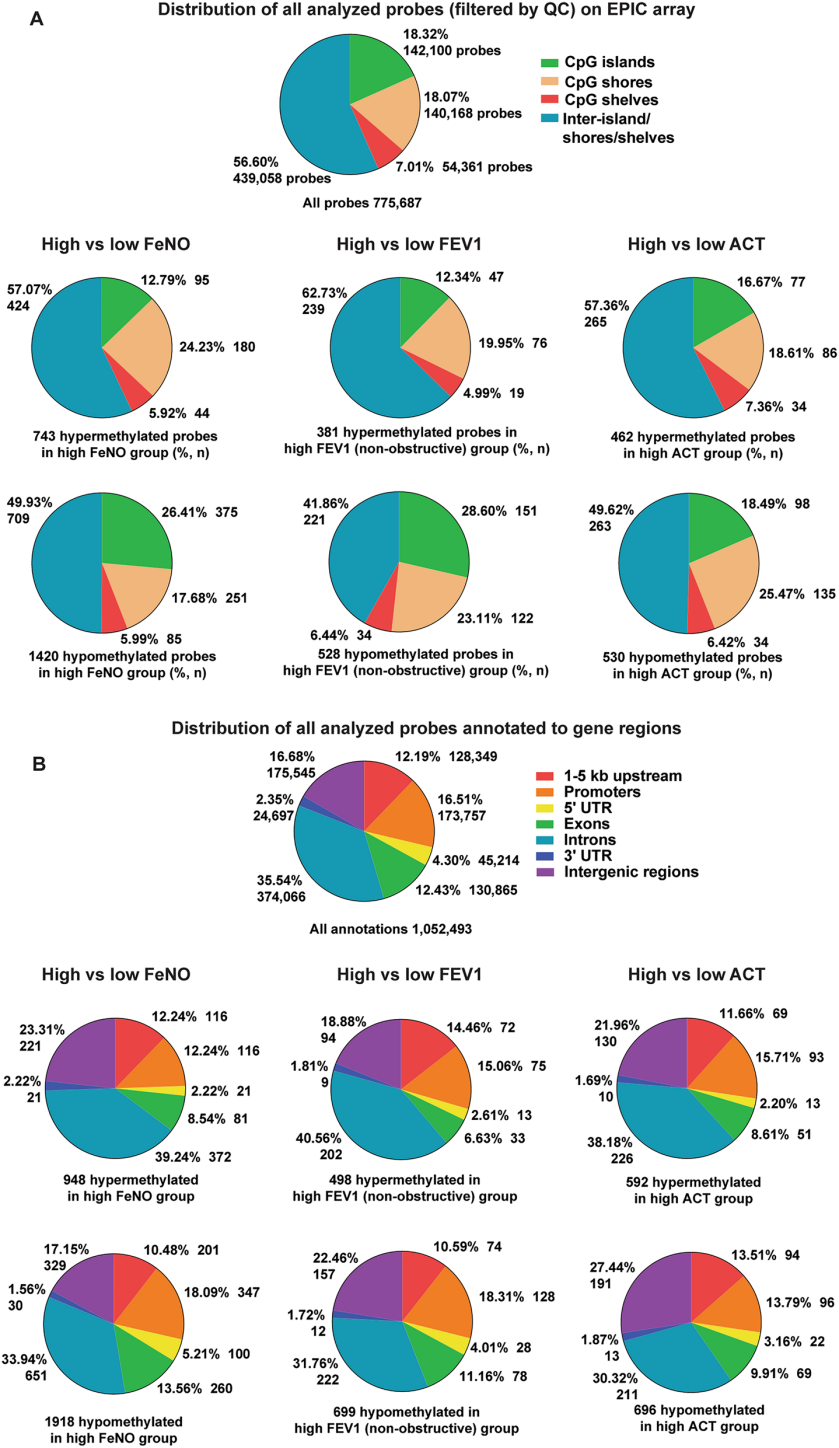
The distribution of differentially methylated CpG probes by genic regions for all three clinical comparisons. (**A**) The CpG sites for all Epic array probes used in our analysis (filtered by quality control [QC]) were annotated to either CpG islands, shores, shelves, or inter-island/shelves/shores, and their distribution (percentage, number of probes) is shown. The hypermethylated and hypomethylated probes for each comparison (FeNO, FEV1, and ACT) were annotated to either CpG islands, shores, shelves, or inter-island/shelves/shores, and their relative distribution (percentage, number of probes) is shown below. (**B**) The CpG sites for all Epic array probes used in our analysis (filtered by QC) were annotated to being in 1–5 kb upstream, promoter, 5’-untranslated region (UTR), exon, intron, 3’-UTR, or intergenic regions, and their distribution (percentage, number of probes) is shown. Some individual probes were annotated to multiple regions of different genes. The hypermethylated and hypomethylated probes for each comparison (FeNO, FEV1, and ACT) were annotated to these genic (1–5 kb upstream, promoter, 5’-UTR, exon, intron, 3’-UTR) or intergenic regions, and their relative distribution (percentage, number of probes) is shown below

The differentially methylated probes or CpG sites for each comparison were next annotated by location within the genome and whether they were located within CpG islands, shores, or shelves. Hypermethylation in CpG islands is often associated with suppression of gene expression. Compared to the low FeNO group, the high FeNO group demonstrated a smaller proportion of hypermethylated and a larger proportion of hypomethylated CpG sites within CpG islands (Fig. 3A). Samples from individuals with high FEV1 also had a high proportion of hypomethylated sites among CpG islands and a smaller proportion of hypomethylated sites among inter-CpG islands/shores/shelves. Finally, within the high FeNO group, a smaller proportion of hypermethylated CpG sites were found in promoters, 5-untranslated regions (UTR), and exons, whereas a greater proportion of hypermethylated sites were found within 3’-UTR and intergenic regions (Fig. 3B). A similar pattern was seen among the high FEV1 and high ACT groups, where a smaller proportion of hypermethylated CpG sites were found in promoters, 5’-UTR, and exons and a greater proportion of hypermethylated CpG sites were found among introns and intergenic regions. Together, these data suggest that the differential DNA methylation is not distributed uniformly throughout the genome, but instead, in select regions of certain genes that may ultimately influence the regulation of those genes.

### Enrichment of pathways from differentially methylated genes

**Fig. 4:**
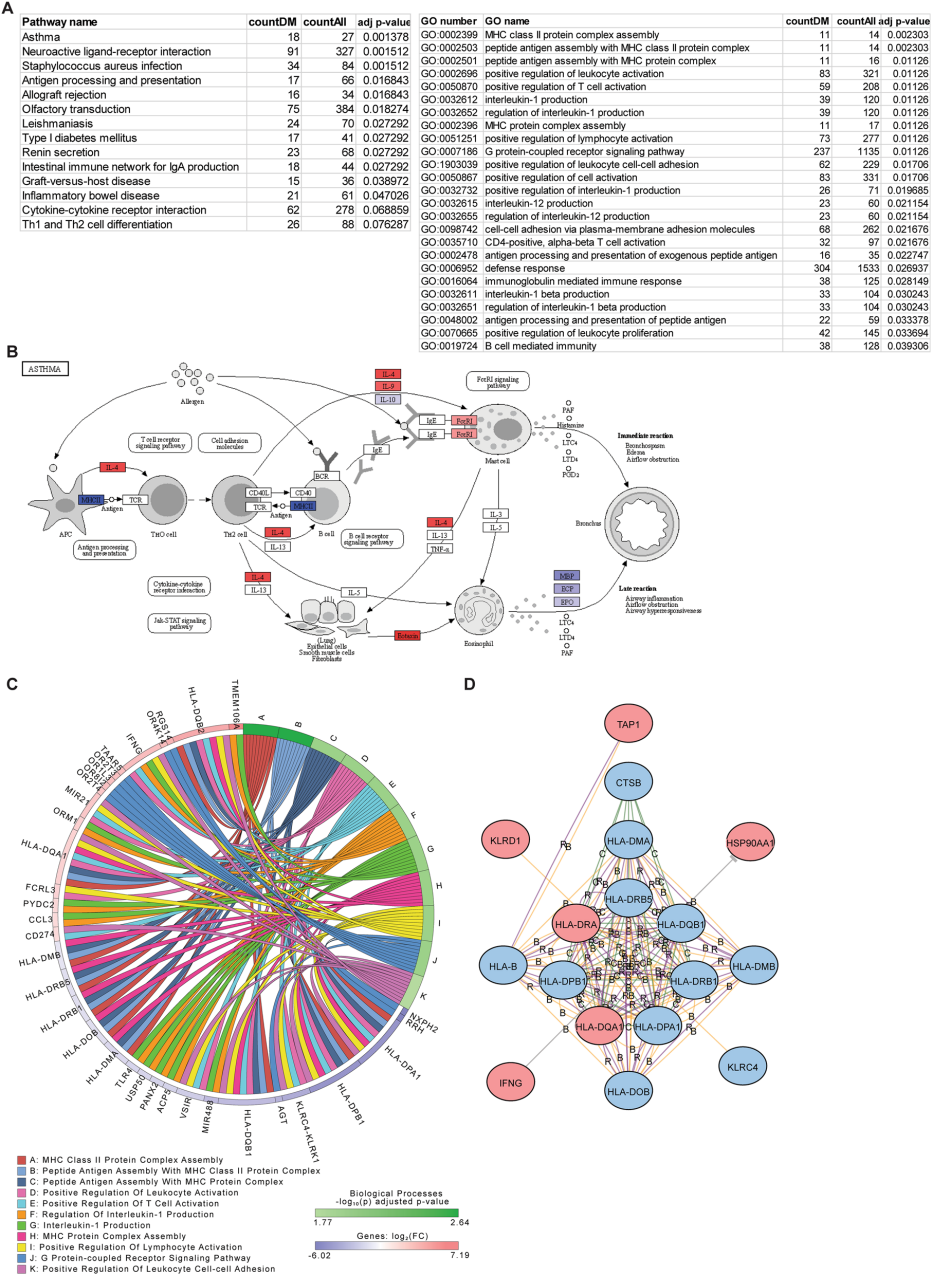
Pathway and gene ontology (GO) enrichment analysis of differentially methylated genes in the high vs low FeNO comparison. (**A**) The differentially methylated CpG sites in the high vs low FeNO comparison were annotated to genes and enriched Kyoto Encyclopedia of Genes and Genomes (KEGG) pathways and GO terms by biologic function were identified using iPathway analysis. Tables list number of differentially methylated genes (countDM) relative to all genes (countAll) that define the pathway or GO term and the adjusted (adj) *p* value (accounting for false discovery rate < 0.05). (**B**) Diagram of the KEGG pathway for “Asthma” with hypermethylated (red) and hypomethylated (blue) genes are shown. (**C**) Shown is a chord diagram of the top enriched biological processes by adjusted *p* value and the common hypermethylated (red) and hypomethylated (blue) genes among them. (**D**) Shown is a network analysis of the hypermethylated (red) and hypomethylated (blue) genes within the GO term “Antigen processing and presentation of peptide antigen”. Interactions are defined as B, binding; C, catalysis; or R, reaction (color figure online)

As each of these CpG sites or probes was annotated to genes, we next examined the functional relevance of these genes and enrichment of functional pathways that were differentially methylated in each comparison. As each gene often had multiple CpG probes, we took the average methylation difference among all probes with adjusted *p* value < 0.05 to determine whether genes were hyper- or hypomethylated. Using iPathway, we found that some of the most highly enriched KEGG pathways among the high vs low FeNO differentially methylated genes include “Asthma”, “Antigen processing and presentation”, “Cytokine-cytokine receptor interaction”, and “Th1 and Th2 cell differentiation” (Fig. 4A). Figure 4B highlights some of the differentially methylated genes within the “Asthma” KEGG pathway and include IL-4, eotaxin, and major histocompatibility complex (MHC) class II genes. GO analysis further revealed enrichment in molecular functions related to MHC class II protein assembly, T cell activation, IL-1 production, and leukocyte adhesion (Fig. 4A). Common differentially methylated genes found among these GO terms include certain *HLA* family genes, *TLR4*, and *IFNG* (Fig. 4C). The relationship between these genes can be further viewed by network analysis (Fig. 4D). iPathway also identified differential methylation of upstream mediators such as *PTPRC* that might regulate other genes downstream (Additional file 1: Figure S4A) or differential methylation of a family of genes including olfactory receptors (Additional file 1: Figure S4B), some of which have been implicated in asthma [24, 25]. Finally, other enriched pathways, such as “neuroactive ligand-receptor interaction”, which may on the surface seem less relevant, include differentially methylated genes such as *P2RY11* (part of the purinergic pathway), *AGT*, and *NMUR2* that are centered in network hubs (Additional file 1: Figure S4D); these genes have also been implicated in other studies as important in asthma [26–29].

**Fig. 5:**
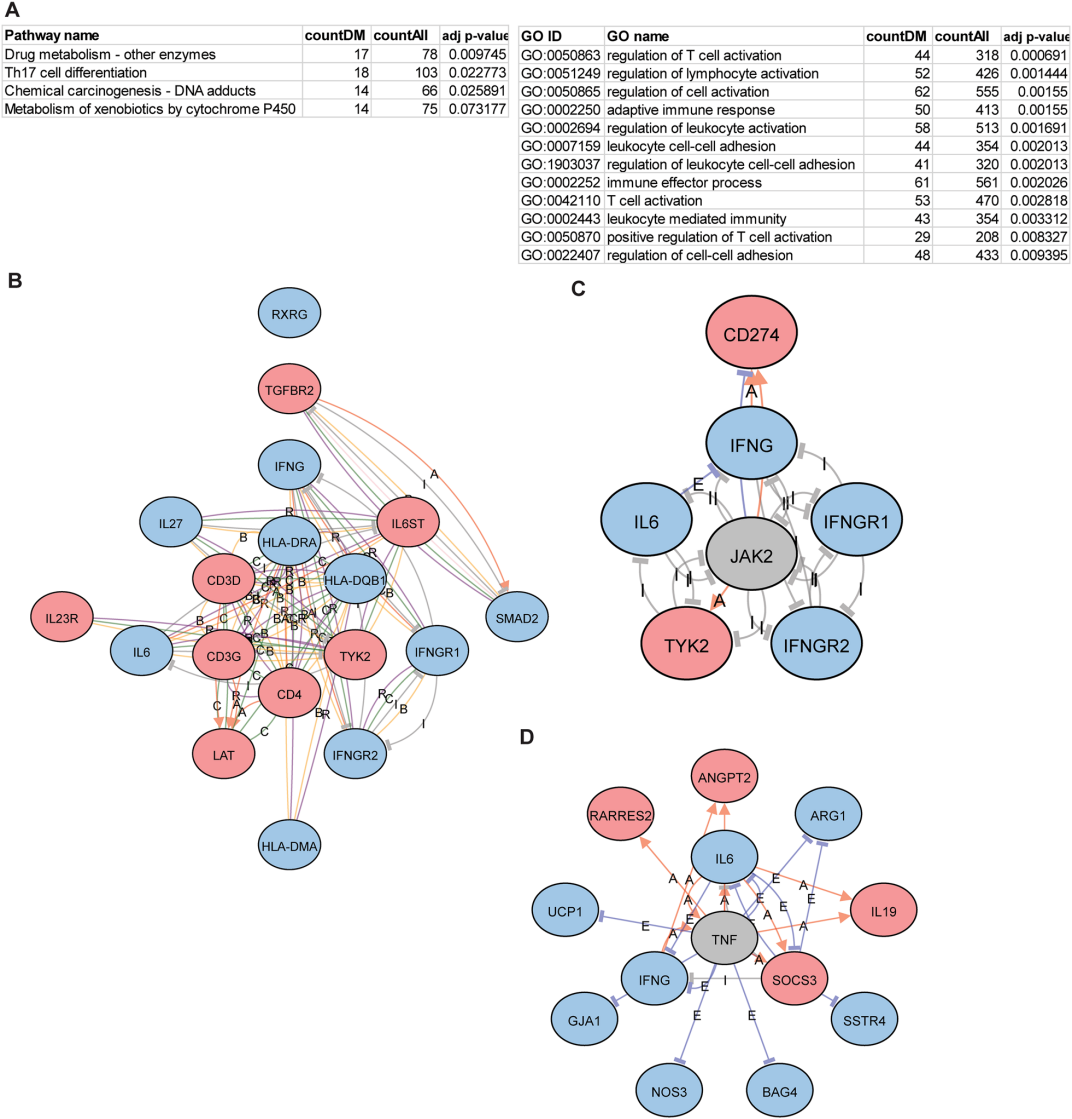
Pathway and GO enrichment analysis of differentially methylated genes in the high vs low FEV1 comparison. (**A**) Differentially methylated genes in the high vs low FEV1 comparison were analyzed by iPathway to identify enriched pathways and GO terms. (**B-D**) Networks from hypermethylated (red) and hypomethylated (blue) genes were generated from the enriched KEGG pathway, “Th17 cell differentiation” (**B**), and from the upstream regulators JAK2 (**C**) and TNF (**D**). Interactions are defined as A, activation; B, binding; C, catalysis; E, expression; I, inhibition; R, reaction (color figure online)

When performing pathway analysis of differentially methylated genes between high vs low FEV1 comparison, different pathways compared to that found in the FeNO comparisons were noted (Fig. 5A). That these pathways were different was not surprising, as most of the differentially methylated CpG sites found in the high vs low FEV1 comparison were different than that found in the FeNO comparison (Fig. 2D). Some of these pathways including “Drug metabolism – other enzymes”, “Chemical carcinogenesis – DNA adducts”, and “metabolism by xenobiotics by cytochrome P450”, which include genes involved in cytochrome P450 metabolism (e.g., *CYP1A1*, *CYP2E1*, *CYP2A13*), glutathione metabolism (*GST* family genes), and glucuronidation (*UGT* family of genes); these pathways have all reported to have some role in asthma [30–33]. Other enriched pathways and GO terms continue to focus on T cell functions including “Th17 cell differentiation”, “Regulation of T cell activation”, “Adaptive immune response”, “leukocyte cell–cell adhesion”, and “T cell activation”. Although these GO terms and pathway names bear similarities to that found in the FeNO comparison group, the differentially methylated genes within these pathways were different, including several genes involved in Th17 differentiation (Fig. 5B and Additional file 1: Figure S5A). Diverse chemokines (*CXCL10, CCR2, CCR10, IL6, IFNG*) and complement (*C3* and *C4A*) genes were also more prominently enriched in this comparison as compared to the FeNO comparison. Finally, iPathway identified *JAK2* and *TNF* as upstream regulators of many of these differentially methylated genes (Fig. 5C and D).

**Fig. 6:**
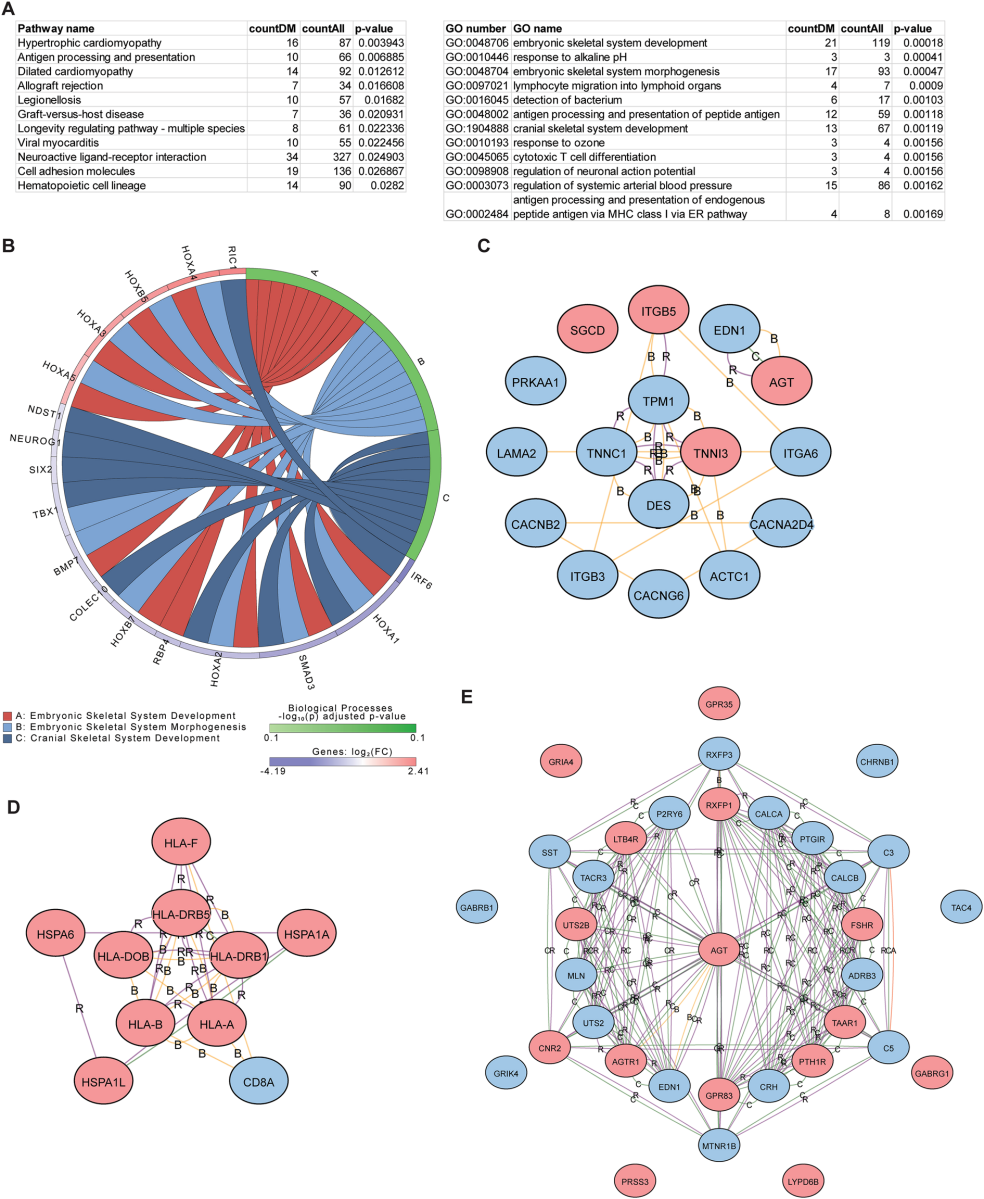
Pathway and GO enrichment analysis of differentially methylated genes in the high vs low ACT comparison. (**A**) Differentially methylated genes in the high vs low ACT comparison were analyzed by iPathway to identify enriched pathways and GO terms. (**B**) Chord diagram was constructed based on enriched GO terms and common hypermethylated (red) and hypomethylated (blue) genes. (**C**–**E**) Network analysis was constructed based on hypermethylated (red) and hypomethylated (blue) genes within the pathways “hypertrophic cardiomyopathy” (**C**), “neuroactive ligand receptor” (**D**), and “antigen processing and presentation” (**E**). Interactions are defined as A, activation; B, binding; C, catalysis; or R, reaction (color figure online)

Pathway analysis of the differentially methylated genes associated with the high vs low ACT comparison identified pathways and enriched GO terms that were, yet again, different from those in the FeNO and FEV1 comparisons. These include KEGG pathways such as “Hypertrophic cardiomyopathy”, “Cell adhesion molecules”, and “Hematopoietic cell lineage” as well as GO terms such as “Embryonic skeletal development” and “Embyronic skeletal system morphogenesis” (Fig. 6A). These latter GO terms particularly highlight developmental genes within the *HOX* family and *SMAD3* as differentially methylated (Fig. 6B). Although the KEGG pathway “Hypertrophic cardiomyopathy” at first glance would seem unusual for a study investigating T cells in asthma, many of the highlighted genes include integrins (*ITGB5*, *ITGA6*), desmin (*DES*), endothelin (*EDN1*), and angiotensinogen (*AGT*) (Fig. 6C) that have all been implicated in asthma or airway remodeling [29, 34–37]. Other pathways such as “Antigen processing and presentation” and “Graft-versus-host disease” and GO terms such as “Antigen processing” were like those highlighted as differentially methylated in the FeNO and FEV1 comparisons (Fig. 6D and Additional file 1: Figure S6). Finally, the high vs low ACT comparison also identified novel genes that form interrelated networks (Fig. 6E).

### Common genes, networks, and pathways among all clinical comparisons

**Fig. 7:**
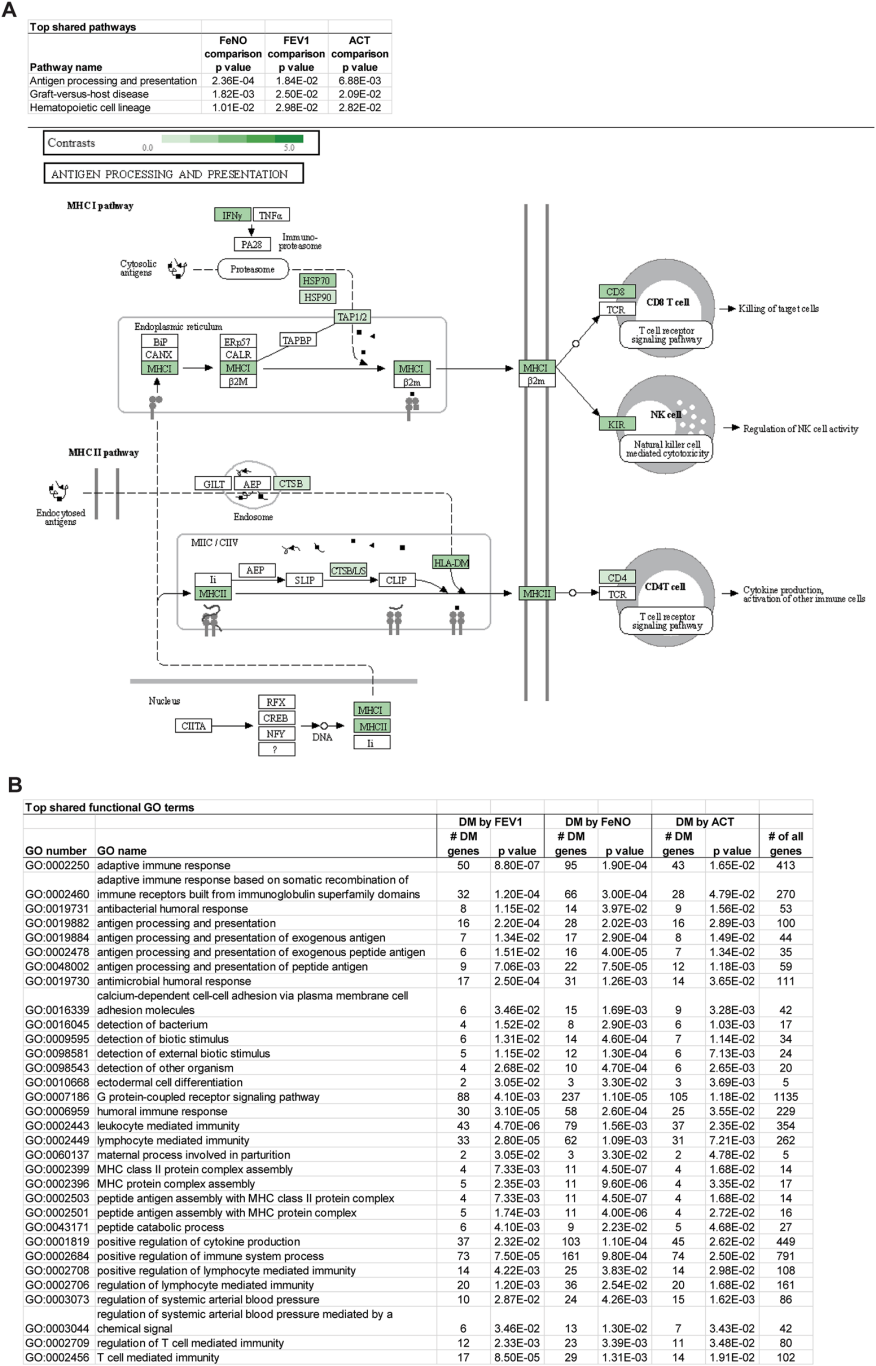
Overlapping pathways and GO terms among the three clinical comparisons. (**A**, **B**) Genes that were differentially methylated between high and low FeNO, FEV1, and ACT comparisons were analyzed by iPathway analysis and those KEGG pathways (**A**) and GO terms (**B**) that were common among the three comparisons are shown in the tables. The pathway and the differentially methylated genes that highlight the pathway for “antigen processing and presentation” are shown in (**A**)

Although comparisons between high vs low FeNO, FEV1, and ACT produced mostly distinct sets of differentially methylated CpG sites and genes, several CpG sites and genes that were consistently found to be differentially methylation among all clinical comparisons. As noted in Fig. 2, there were 42 probes in common that were differentially methylated among all comparisons. When clinical severity and directionality of methylation changes were considered, 13 probes were hypermethylated in the more severe or more symptomatic group (high FeNO, low FEV1, low ACT) and 11 probes were hypermethylated in the less severe, less symptomatic group (Fig. 2E). Since genes typically have multiple CpG sites and probes, one can also examine the genes, differentially methylated in at least one probe or CpG site, that were in common in all comparisons. Analysis of those genes revealed three pathways and several GO terms, almost all of which focus on antigen processing and presentation and T cell function, that stand out (Fig. 7). Many of these genes include genes of the HLA family, and upstream mediators including protein tyrosine phosphatase receptor type C (*PTPRC*) and type J (*PTPRJ*) that are known regulators of B and T cell antigen signaling [38, 39].

## Discussion

In this study of adults with variable asthma severity, we identified extensive genome-wide differences in DNA methylation in CD3 + T cells of individuals with asthma that correlated with markers of airway inflammation, lung function, and asthma control. The differences in DNA methylation were widespread throughout the genome but varied depending on the comparison. Many of the genes found to be differentially methylated are known to be involved in antigen presentation or modulation of T cell differentiation, supporting the potential importance of DNA methylation changes in asthma. Although some genes have been shown in the literature to be clearly associated with asthma, other genes are novel, potentially providing new insights into asthma pathophysiology.

Changes in DNA methylation have been well-described in children who develop asthma [1, 2, 5, 8] and represent a fundamental mechanism that support the fetal-origin hypothesis of the disease. However, asthma is a complex disease where patients develop asthma at different ages of onset and exhibit varying levels of airway inflammation and severity. Although changes in DNA methylation are well-associated with asthma development [1–3, 8, 12], the role of DNA methylation in modulating asthma severity is less appreciated. By examining multiple facets of asthma severity including FeNO, pre-bronchodilator FEV1, and symptom control by ACT, we were able to identify methylomic changes that were associated with each of these clinical variables. That methylation differences with each of these comparisons produced a unique set of differentially methylated probes and genes speaks to how each of these variables—FeNO, FEV1, and ACT—serve as different measures of asthma severity and pathophysiology. That many of the identified differentially methylated genes were different from those described in studies of children highlights potential differences between adult and childhood asthma pathophysiology and the role methylation plays in asthma development vs severity.

As compared to many other DNA methylomic studies in asthma that utilize whole blood or peripheral blood mononuclear cells, we specifically isolated CD3 + T cells for our study, as DNA methylation patterns are often cell-type specific and identifying changes in methylation attributable to a specific cell type may provide greater insights into the significance of these changes. Because anti-CD3 + magnetic bead isolation is subject to variability in technique, we additionally analyzed samples by cell-type deconvolution, which showed that although our samples consisted mostly of CD4 and CD8 T cells, a small percentage included NK cells, B cells, monocytes, and neutrophils. Our model thus used cell-type deconvolution as a covariate. Although other reference-free methods for cell-type deconvolution [40] could also have been used, the combination of CD3 + magnetic bead isolation and the cell-type deconvolution we employed was able to identify methylation differences with genes involved in antigen presentation and T cell function and activation. More specifically, methylation differences between those with high and low FeNO, which is often used clinically as a marker of type 2 airway inflammation, were associated with methylation differences in *IL4*, *CCL11* (eotaxin), and many HLA genes that are involved in antigen presentation and MHC class II assembly. Coincidentally, the cutoffs between subjects with high vs low FeNO in our cohort fell near 25 ppb, which is the cutoff commonly used in clinical guidelines [41] to distinguish type 2 vs non-type 2 asthma, suggesting that these methylation differences may be important in distinguishing these two types of asthma. The lack of available data on subjects’ peripheral eosinophil levels, total IgE level, or specific aeroallergen sensitivity testing is, however, a limitation and prevents extrapolation of our findings from FeNO to a broader “type 2 high” phenotype.

Although comparisons of methylation patterns between subjects with high vs low FEV1 also resulted in methylation differences in genes associated with T cell function and activation, there were also other genes that were differentially methylated, including many related to drug metabolism and cytochrome P450. The significance of this is unknown but may indicate a mechanism by which environmental toxins can affect asthma and influence lung function. Other genes identified as differentially methylated in the high vs low FEV1 comparison include those involved in inflammation and are downstream targets of *JAK2* and *TNF*. These include genes such as *IL6*, interferon γ (*IFNG*), *SOCS3*, and *NOS3*. Complement genes (*C3* and *C4A*) and genes involved in TH17 differentiation were also highlighted. These findings indicate how much methylation differences in these genes that regulate and modulate inflammation may influence lung function. Finally, it was interesting to note that individuals with asthma with different levels of symptom control, as measured by ACT, had differential methylation in yet other genes, some of which were involved in development and airway remodeling, such as HOX family genes, integrins (*ITGB5*, *ITGA6*), desmin (*DES*), and endothelin (*EDN1*), suggesting that differential methylation of these genes may have an important role in daily symptoms and asthma control. As asthma patients with diminished lung function and persistent symptoms often exhibit pathologic evidence of airway remodeling, the differential methylation of these inflammatory and developmental genes may predispose patients to developing airway remodeling.

Some of the genes that were differentially methylated were observed to be differentially methylated in other studies of asthma as well, such as *RUNX3* and *IL4* [8]. This supports the generalizability of our findings. Certain genes, however, were found unique to our study but differentially methylated across all our comparisons. These include certain HLA family genes, *PTPRC*, and *PTPRJ*. These latter two genes are protein tyrosine phosphatases that have been described as master regulators that are differentially expressed in transcriptomic studies of children with severe asthma [39, 42]. These genes have also been shown by others to be regulated by DNA methylation and our study is the first to identify the differential methylation of these genes to be associated with asthma severity. *AGT*, which codes for angiotensinogen, was another gene present in the center of many of our network analyses and shown among all our clinical comparisons as differentially methylated.

Our study was unique in that we studied a cohort of asthma patients from China, as compared to other population-based DNA methylomic studies that focus on European or North American cohorts. Differences in local environment and ancestral origin may account for some of the differences in DNA methylation in our study compared to others though there were many genes and pathways that were also similar. We excluded patients with smoking history to eliminate potential confounding effects of patients with COPD or asthma-COPD overlap. Although the total size of our cohort was small, longitudinal collection of blood samples from the same patient enhanced the power of our study and all analyses were adjusted for repeat measurements from the same patient.

One of the limitations of the study was that we did not observe robust longitudinal changes in DNA methylation that correlated with a given subject’s FEV1, FeNO, or ACT over time, as we initially set out to do. Instead, the DNA methylation patterns for each patient remained relatively stable, and greater variations in DNA methylation were present between patients as compared to changes in methylation over time within subjects. This suggests that DNA methylation may be a stable epigenetic mark though this may also be a consequence of the fact that the degree of asthma severity in most of the subjects remained relatively stable throughout longitudinal follow-up and no subjects developed acute exacerbations during the study period. We thus pivoted from the original goal of the study and instead, focused our analyses on correlating differences in DNA methylation with subjects’ average FeNO, FEV1, or ACT over time. Even though this secondary analysis is viewed as a limitation of this study especially in light of a small cohort with potential for being underpowered, the cohort consisted of diverse subjects with varying baseline asthma severity, so we were still able to identify statistically significant differences in DNA methylation that correlated with meaningful clinical markers of asthma severity. We used several approaches in our analysis, including modeling FeNO, FEV1, and ACT as continuous variables or dividing them into tertiles (top, middle, and bottom third), and found that the strongest correlations with methylation occurred when subjects were divided into dichotomous groups of high vs low FeNO, FEV1, or ACT. This is potentially because biological effects were best observed with subjects at the extreme ends of FeNO, FEV1, and ACT, and minor variations in day-to-day measures of FeNO or FEV1 could result in noise that make it difficult to correlate methylation with measures of FeNO or FEV1 when viewed as continuous variables. A larger cohort would improve the power of the study and potentially allow us to make stronger correlations between methylation and these clinical indices or identify even more differentially methylated genes associated with high or low FeNO, FEV1, or ACT. Future studies with a larger cohort that includes subjects with more variable asthma control will allow us to also examine whether DNA methylation might change over time in patients with labile disease. Nonetheless, the fact that we were able to identify methylation differences even within a small cohort emphasizes the potential importance of DNA methylation changes that are associated with varying clinical indices of asthma.

Although we observed robust patterns of differential methylation, the biological significance of these differences in methylation is unknown. We did not collect samples to simultaneously analyze for RNA-Seq analysis and thus, could not correlate methylation changes with gene expression changes, which is a major limitation. In our comparisons, we observed shifts in the distribution of methylation differences, with more hypomethylation in CpG islands and shores, for example, among subjects with high FeNO. This suggests that there may be an increase in gene expression among patient with high FeNO, which would be congruent with the presence of greater airway inflammation, though this remains to be determined experimentally. To summarize the net effect of methylation at the gene level, we calculated the weighted average of the methylation changes of all CpG sites for a given gene. Calculating the average methylation for a gene has the advantage of avoiding bias that may occur with genes that possess variable number of CpG sites assayed, uneven distribution of CpG sites, and inherent genomic biases within the Epic array. However, the effects of methylation are complex and often context dependent and this approach of taking the weighted average may obscure important methylation differences that only occur in specific genomic regions. Furthermore, this approach does not completely remove bias from genes that possess a greater number of CpG sites and sequencing-based approaches such as whole genome bisulfite sequencing, though more costly, would provide a more comprehensive picture of methylation differences. DNA methylation is only one epigenetic mechanism, and other epigenetic mechanisms, including histone modifications [43] and noncoding RNAs [44, 45] that are also recognized to be important in asthma pathogenesis, were not examined in this study. Gene pathway enrichment analysis was performed over the background of all genes analyzed by the Epic array, but ultimately, the functional consequences of the observed methylation changes are unknown. The genes, networks, and pathways that we highlight in this study require future studies to determine their mechanistic relevance.

In conclusion, differential patterns of DNA methylation were observed in the peripheral blood T cells of patients with different degrees of asthma severity. These differences occurred in many genes associated with T cell function, antigen presentation, and leukocyte activation, indicating a potential role for DNA methylation to modulate the immunologic pathways critical to asthma. Novel differentially methylated genes were also identified that may influence T cell function or affect asthma pathobiology and warrant future studies. Together, these data support the potential for this epigenetic mechanism as a critical determinant of asthma severity.

## Supplementary Information

Additional file 1.

## Data Availability

Sequencing data that support the findings of this study have been deposited in the National Center for Biotechnology Information Gene Expression Omnibus database under accession number GSE226257.
