# Supplementary material for: Differences in the DNA methylome of T cells in adults with asthma of varying severity
Source: Clin Epigenetics. 2024 Oct 8;16:139. doi: 10.1186/s13148-024-01750-7 (PMC11459694; doi:10.1186/s13148-024-01750-7)
Supplement: Supplementary file 1 — Additional file 1. [file 13148_2024_1750_MOESM1_ESM.docx]

**Online Data Supplement for:**

**Differences in the DNA Methylome of T cells in Adults With Asthma of Varying Severity**

Yixuan Liao, Raymond J. Cavalcante, Jonathan B. Waller, Furong Deng, Anne M. Scruggs, Yvonne J. Huang, Ulus Atasoy, Yahong Chen, Steven K. Huang

Address correspondence to:

Steven K. Huang, MD Yahong Chen, MD

Division of Pulmonary and Critical Care Medicine Department of Pulmonary and Critical Care Medicine

University of Michigan Peking University Third Hospital

6301 MSRB III No.49, Huayuan North Road

1150 W Medical Center Dr. Haidian District

Ann Arbor, MI 48109 Beijing, China 100191

Phone: (734) 647-6477 Phone: +86-13910232918

Fax: (734) 764-4556

Email: [stehuang@umich.edu](mailto:stehuang@umich.edu) email: [chenyahong@vip.sina.com](mailto:chenyahong@vip.sina.com)





**Figure S1.** Blood sample distribution and cell type deconvolution by DNA methylation analysis. **(A)** Alignment of each subject per subject identification (ID) and the duration of patient follow-up, with symbols representing the dates blood samples were taken from each subject. **(B)** Cell type deconvolution was performed based on the DNA methylation pattern of each sample, with the relative proportion of each cell type shown.


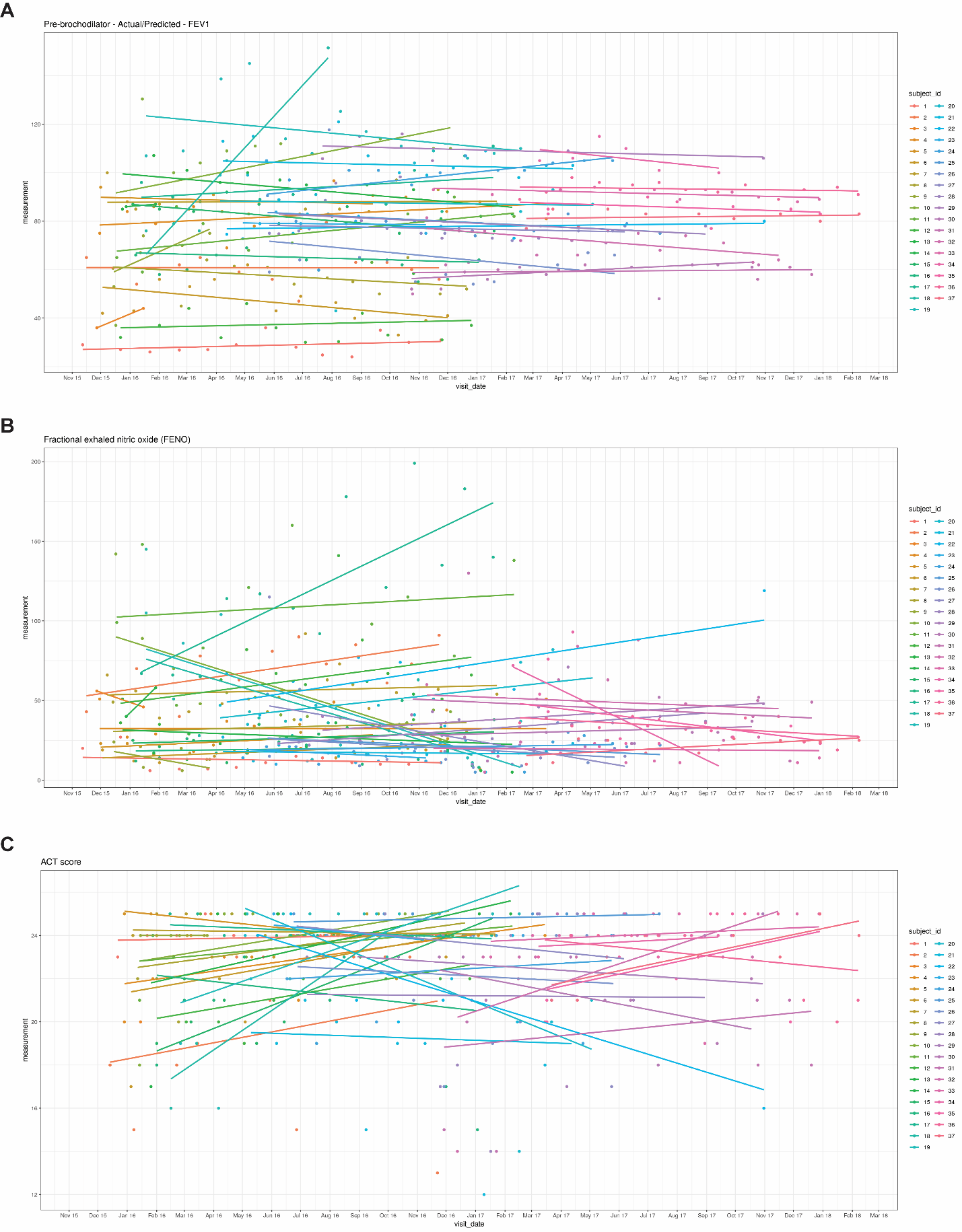


**Figure S2.** Measures of clinical parameters of asthma over time for each subject. **(A)** Pre-bronchodilator forced expiratory volume in 1 second (FEV1) as a percent predicted, **(B)** fraction of exhaled nitric oxide (FeNO) in parts per billion, and **(C)** Asthma Control Test (ACT) score were measured for each subject at each of their visits and values of these clinical parameters are graphed over time and analyzed by linear regression analysis.


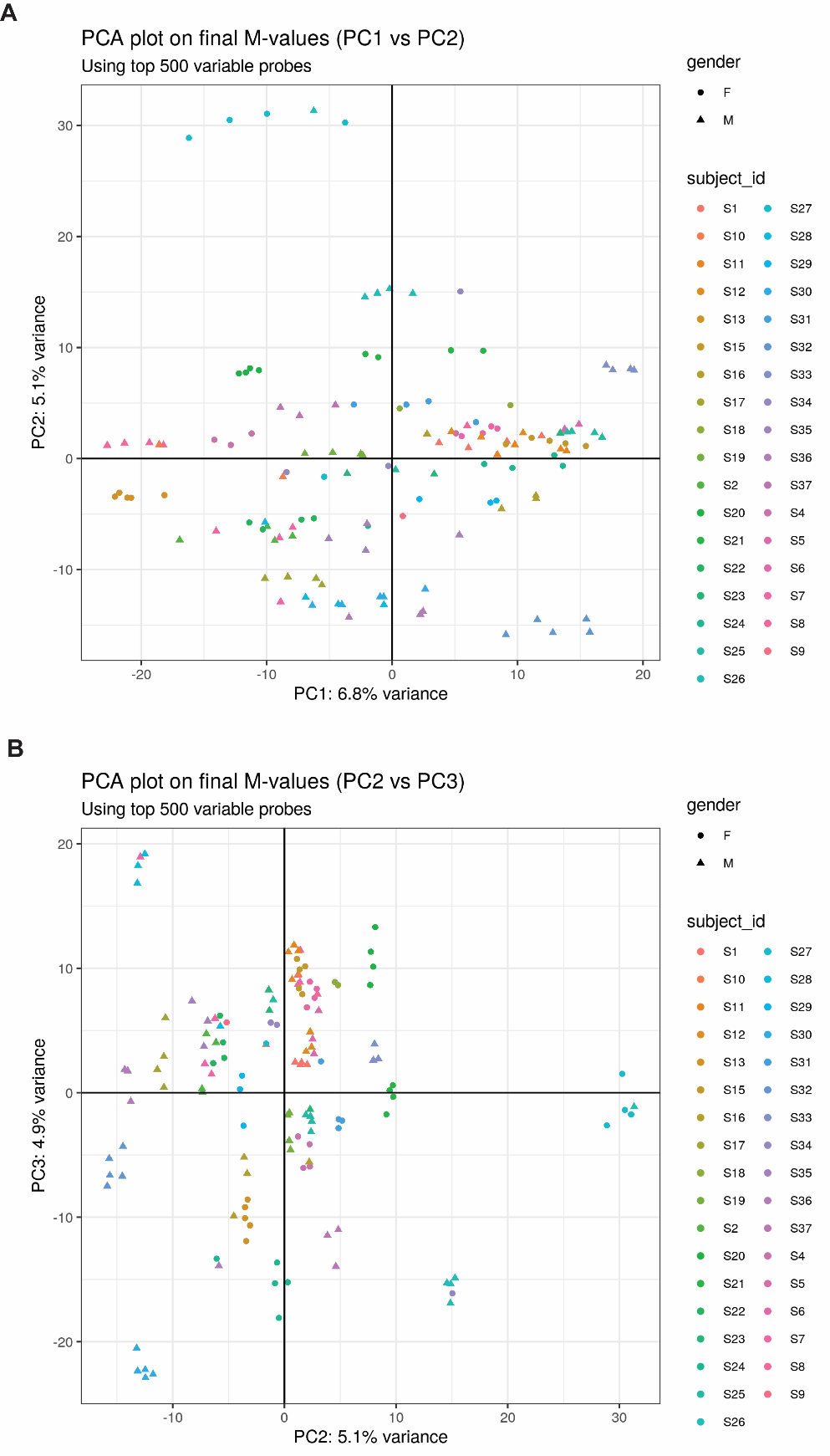


**Figure S3.** Principal component analysis (PCA) plots of patient samples based on DNA methylation of the top 500 most variable probes were constructed, with **(A)** depicting the principal component (PC) variable 1 vs PC2 and **(B)** depicting PC2 vs PC3. Data points of the same color represent repeated samples taken from the same subject (defined by subject_id) over time.


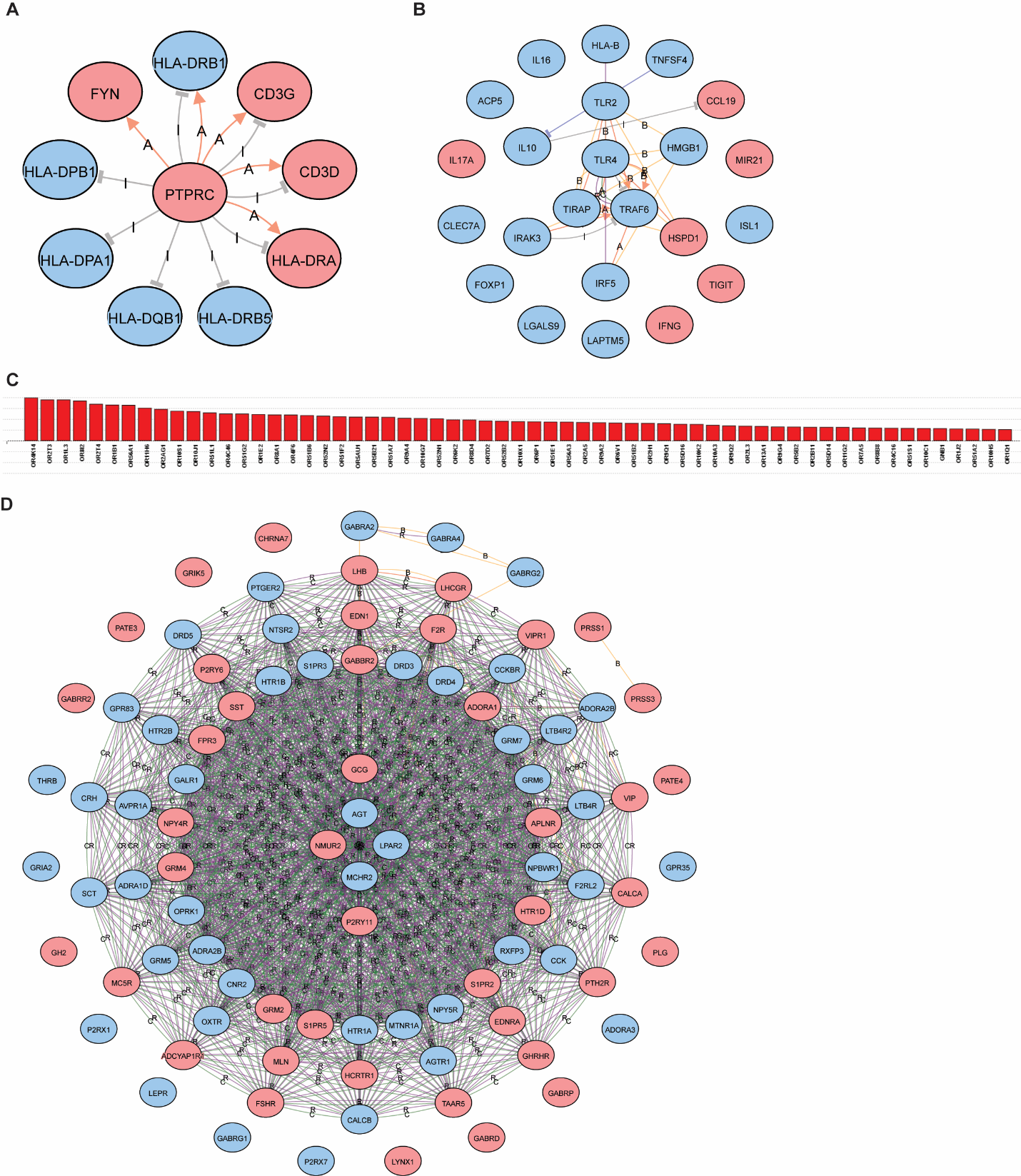


**Figure S4.**Networks from pathways of differentially methylated genes identified between high vs low FeNO comparison. **(A)** *PTPRC* was identified by iPathway as an upstream regulator of several hyper- (red) and hypo- (blue) methylated genes in the dataset. **(B and D)** Network of genes from the gene ontology (GO) pathway “Interleukin-12 production” **(B)** and the Kyoto Encyclopedia of Genes and Genomes (KEGG) pathway “Neuroactive ligand-receptor interaction” **(D)** that were differentially methylated in the dataset. Interactions are defined as A, activation; I, inhibition; B, binding; C, catalysis; R, reaction. **(C)** Graphical representation of hypermethylated genes from the “Olfactory receptor” pathway.

**
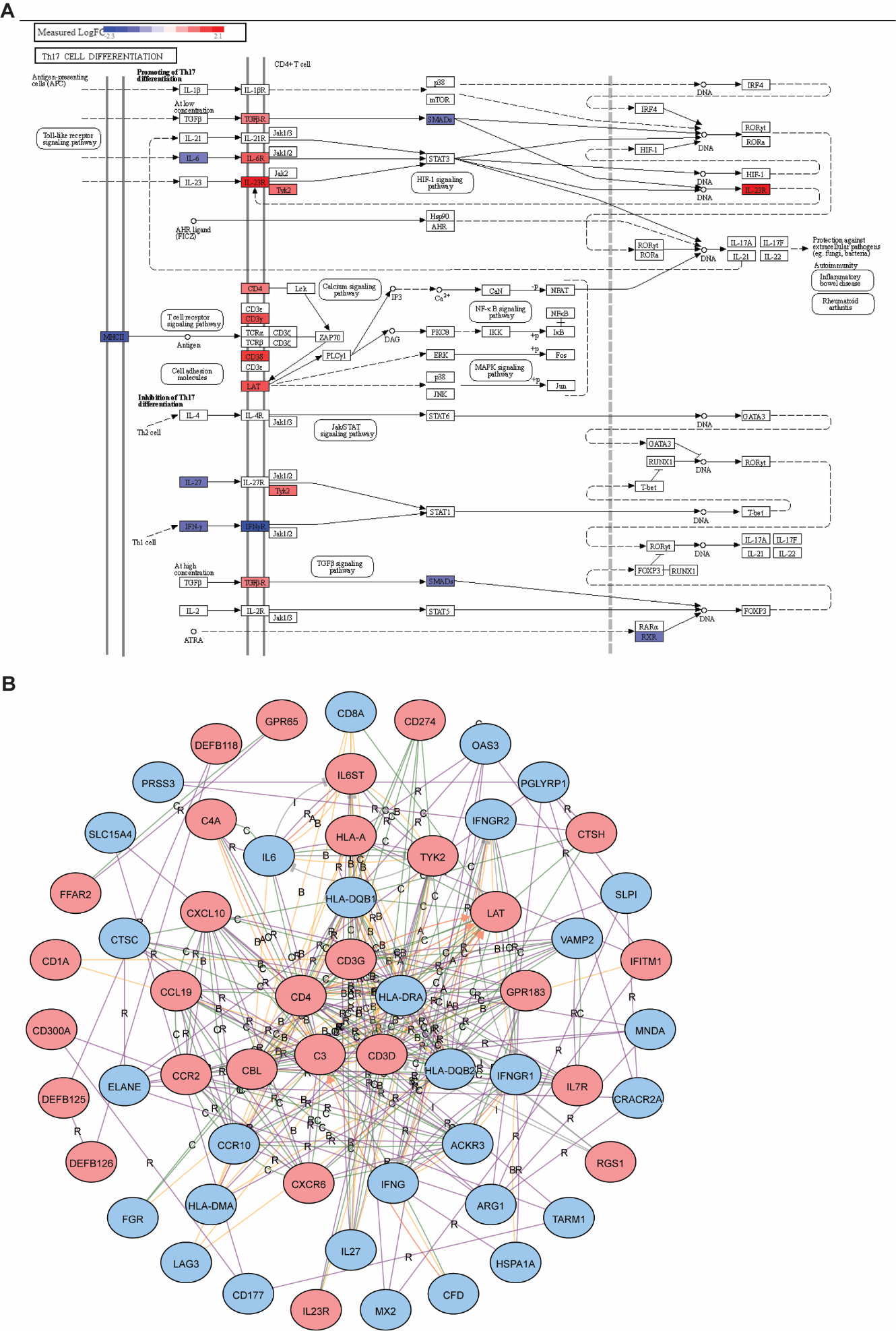
**

**Figure S5.** Differentially methylated genes identified in the high vs low FEV1 comparison. **(A)** The KEGG pathway “Th17 cell differentiation” was identified as enriched among the differentially methylated genes in the high vs low FEV1 comparison. **(B)** A network was generated from genes in the GO category “Immune response” that were differentially methylated. Hyper- (red) and hypo- (blue) genes are indicated. Interactions are defined as A, activation; I, inhibition; B, binding; C, catalysis; R, reaction.


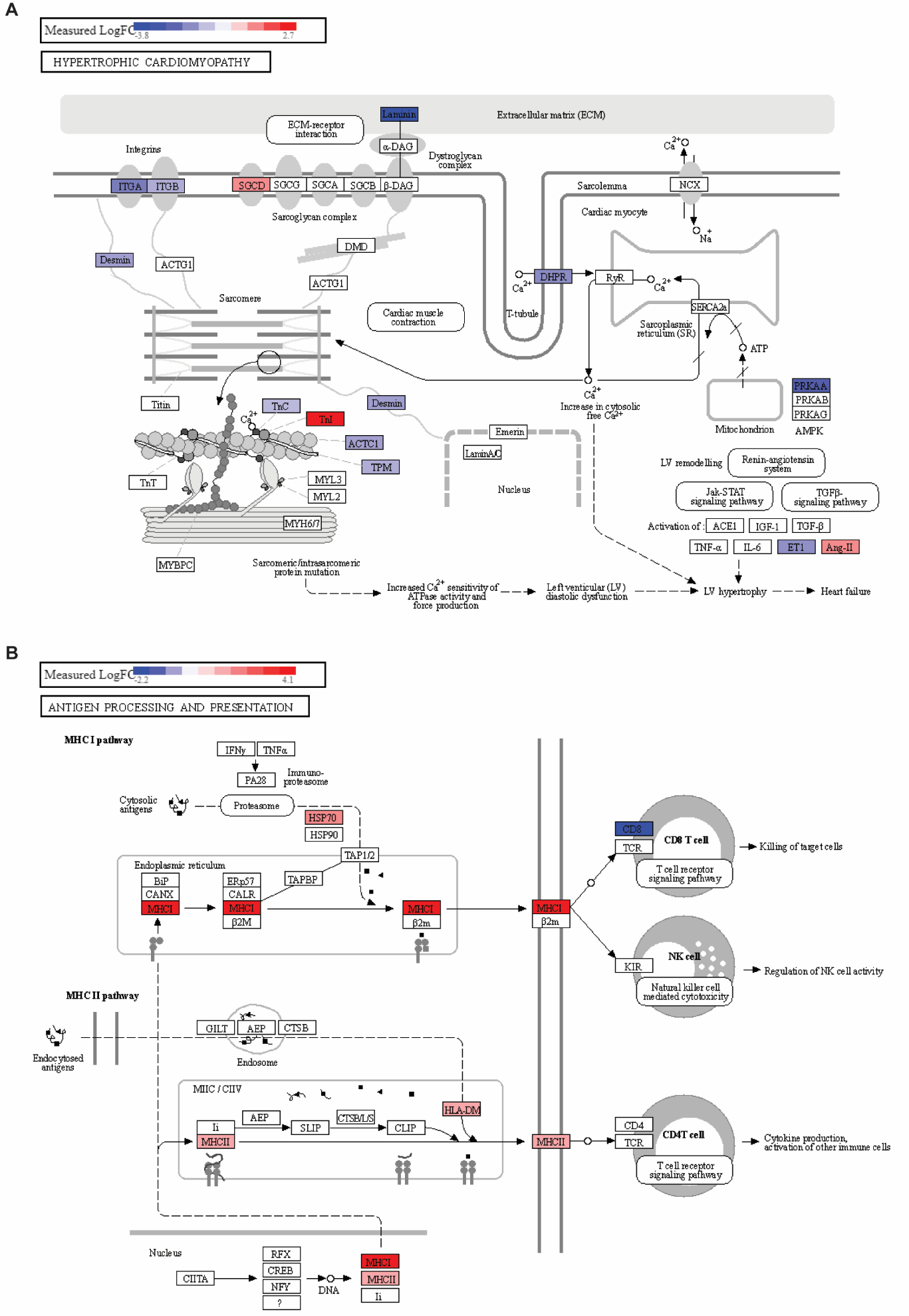


**Figure S6.** Enriched KEGG pathways among the differentially methylated genes in the high vs low ACT comparison. **(A and B)** The KEGG pathway “Hypertrophic cardiomyopathy” **(A)** and “Antigen processing and presentation” **(B)** were the top two pathways among the differentially methylated genes within the high vs low ACT comparison, with red representing hypermethylated and blue representing hypomethylated genes.
